# How does evolution tune biological noise?

**DOI:** 10.3389/fgene.2014.00374

**Published:** 2014-10-28

**Authors:** Magali Richard, Gaël Yvert

**Affiliations:** Laboratoire de Biologie Moléculaire de la Cellule, Ecole Normale Supérieure de Lyon, Centre National de la Recherche Scientifique – Université de LyonLyon, France

**Keywords:** stochasticity, evolution, bet-hedging, nucleosome, phenotypic buffering

## Abstract

Part of molecular and phenotypic differences between individual cells, between body parts, or between individuals can result from biological noise. This source of variation is becoming more and more apparent thanks to the recent advances in dynamic imaging and single-cell analysis. Some of these studies showed that the link between genotype and phenotype is not strictly deterministic. Mutations can change various statistical properties of a biochemical reaction, and thereby the probability of a trait outcome. The fact that they can modulate phenotypic noise brings up an intriguing question: how may selection act on these mutations? In this review, we approach this question by first covering the evidence that biological noise is under genetic control and therefore a substrate for evolution. We then sequentially inspect the possibilities of negative, neutral, and positive selection for mutations increasing biological noise. Finally, we hypothesize on the specific case of H2A.Z, which was shown to both buffer phenotypic noise and modulate transcriptional efficiency.

The recent advances in dynamic imaging and single-cell studies have revealed the stochastic nature of biochemical reactions. Numerous factors are known to affect the degree of noise in these reactions, including temperature (Jo et al., 2005), drug treatment (Dar et al., 2014), age (Bahar et al., 2006) and, very importantly, genotypes (Raser and O’Shea, 2004; Levy and Siegal, 2008; Ansel et al., 2008; Hornung et al., 2012). If mutations can modulate a reaction without necessarily changing the average concentration of its product, then they do not fit in the traditional (often deterministic) view of genotype–phenotype control. Such mutations can change the probabilistic laws of single-cell traits, such as phenotypic noise, which may have important consequences at the multicellular level (Yvert, 2014). Noise has the property to increase disorder. In contrast, living systems are highly organized, developmental processes are under many constrains, and numerous phenotypic traits display robustness to stochastic variation. It is therefore unclear how optimization and control of noise can affect both fidelity and diversity. One way to apprehend this is to examine the mutations that were shown to increase or decrease noise levels. In this review, we first present evidence that noise is under genetic control. We then speculate on the ways by which natural selection acts on it. Finally, we hypothesize on the contribution of histone variant H2A.Z to noise evolution.

## MOLECULAR NOISE IS UNDER GENETIC CONTROL

A wealth of information on molecular noise has been gathered by the study of gene expression. Tracking fluorescent reporters in single cells revealed the stochastic nature of gene expression (Elowitz et al., 2002) and identified mutations that modulate noise in protein abundance. First, changing the number of copies of a gene affects its noise level. Several studies showed that noise scaled with the invert root of copy number and this property was even used as a tool to separately estimate intrinsic and extrinsic noise (Volfson et al., 2006; Stewart-Ornstein et al., 2012). Thus, copy number variations which are abundant in natural populations (Katju and Bergthorsson, 2013) are a likely source of noise modulation. Secondly, changing the location of a gene can also change its expression noise. This was illustrated when comparing two integration sites of a reporter system in yeast (Becskei et al., 2005). It was also later observed when integrating a reporter system in chicken cells (Viñuelas et al., 2013). Thus, genetic translocations are another possible way to modulate noise in gene expression in natural populations. Consistently, mutations in chromatin modifying enzymes, such as yeast SAGA, INO80, or SWI/SNF, increased noise (Raser and O’Shea, 2004) and mutations in several HDAC complexes were also reported to do so (Weinberger et al., 2012). Remarkably, deletion of chromatin-binding factor Sir1 caused stochastic release of silencing at one of two yeast loci (*HML* or *HMR*), thereby generating cellular states epigenetically transmitted to daughter cells (Pillus and Rine, 1989; Xu et al., 2006). Thus, genes encoding chromatin modifiers are possible mutational targets for modulating expression noise of other genes through evolution.

Another way to evolve gene expression noise is to alter the sequence of a promoter region. For instance, yeast genes containing a TATA box in their promoter have higher expression noise than average (Zhang et al., 2009) and mutants lacking such TATA box display lower expression noise (Raser and O’Shea, 2004; Blake et al., 2006; Murphy et al., 2007; Hornung et al., 2012). It has also been demonstrated that the number and the location of transcription factor binding sites within a promoter can affect expression noise without changing expression mean (Octavio et al., 2009; To and Maheshri, 2010). Consistently, each target of the yeast Zap1 transcription factor displays a specific scaling of noise versus mean in response to zinc exposure (Carey et al., 2013). Similarly, the sequence of mammalian gene promoters is a primary determinant of the fine-scale dynamics of gene expression bursts (Suter et al., 2011). Accordingly, modifying the promoter of a cell-cycle regulated gene such that a critical transcription factor binding site became occupied by a nucleosome caused an increase in cell–cell variability and impairment of growth fitness (Bai et al., 2010). Perhaps the most direct exploration of the possible evolution of noise levels by mutations in promoter regions is the work of Hornung et al. (2012) who studied libraries of mutated yeast promoters. Two types of mutations (affecting TATA box sequences or generating out-of-frame ATG) significantly modified burst size and noise level, and this effect was characteristic of high-noise promoters. Since most of the promoters tested were insensitive to mutations, the authors suggested that selection might protect promoters from mutations that would affect burst size and therefore expression noise.

All these observations show that there are many possibilities by which expression noise levels can evolve in natural populations. Although the evolution of gene expression has been intensively studied on the basis of a change in mean expression levels, studies on how expression noise evolves within and between species have been very rare. An early investigation by Ansel et al. (2008) showed that expression noise segregates as a complex genetic trait. It was later followed up by Fehrmann et al. (2013) who found that some genetic sources of this noise were natural mutations in transmembrane transporters. Although these studies were based on single-cell measurements, it is also possible to derive similar conclusions by exploring intra-genotype variation of bulk mRNA levels, as shown in plants (Jimenez-Gomez et al., 2011) and humans (Hulse and Cai, 2013). In this case, however, a major difficulty is to properly exclude that the observed variability is caused by hidden factors. For example, Francesconi and Lehner (2014) showed that subtle differences in developmental time between samples could create abundant intra-genotype diversity. Additional studies on the evolution of gene expression noise are needed to understand how and when changes in noise occurred, and whether they were subjected to selection.

## PHENOTYPIC NOISE DIFFERS BETWEEN NATURAL POPULATIONS

Molecular noise does not systematically generate phenotypic noise. There are many ways by which living systems can attenuate input fluctuations so that their output phenotype remains stable (see below). Since evolutionary selection acts at the phenotypic level, it is important to inspect what evidence supports (or not) the evolution of phenotypic noise. A first step in this direction is to investigate whether natural populations display different levels of phenotypic noise. Note that the term “phenotypic noise” relates here to intra-genotype variability, which can result from stochastic processes or unknown environmental variation. For some biological systems, this type of noise can be quantified experimentally. In the yeast *Saccharomyces cerevisiae*, single-cell experiments showed that noise in morphological traits and in cell division time differs between natural strains (Yvert et al., 2013; Ziv et al., 2013). For multicellular systems, recombinant inbred lines offer the possibility to measure phenotypic traits in independent individuals sharing the same genotype. In maize, inter-individual trait variability was shown to differ between lines, and genomic regions associated with this variability could be detected (Ordas et al., 2008). This suggests that phenotypic noise differs among natural populations. This is important because microevolution then has the possibility to act on it.

Investigating phenotypic noise in wild populations is challenging because of the genetic heterogeneity between individuals. It is nonetheless possible to examine if the environmental variance differs between genotypic categories. For example, this was reported for the weight of wild snails breeded in laboratory conditions (Ros et al., 2004). When the same phenotypic trait is duplicated on individuals, such as left and right symmetrical body parts, quantifying intra-individual trait variation is possible. This way, high levels of noise in the fly wing morphology could be fixed by applying artificial selection on a wild population (Carter and Houle, 2011). For some traits, even more than two independent measures are available from a single individual. This is the case for plant seeds. Studies in the wild showed that the variability of germination timing between seeds differed among populations of the desert plant *Plantago insularis* (Clauss and Venable, 2000). It is possible that part of this variability is not due to genetic heterogeneities between seeds but is modulated by the plant genotypic background. Demonstrating this would prove that phenotypic noise differs among natural populations.

Another way to interrogate the evolvability of phenotypic noise is to look for mutations causing or reducing it. In this regard, an interesting example is the genetic perturbation of a signaling cascade in *Bacillus subtillis* that generated noise in the fate (sporulation) of individual cells within a clonal mutant population (Eldar et al., 2009). Other remarkable examples are yeast gene deletions causing elevated cell–cell variability in morphological traits (Levy and Siegal, 2008). These examples revealed that phenotypic noise may evolve by mutating specific gene circuits or by disrupting pleiotropic genes.

**FIGURE 1:**
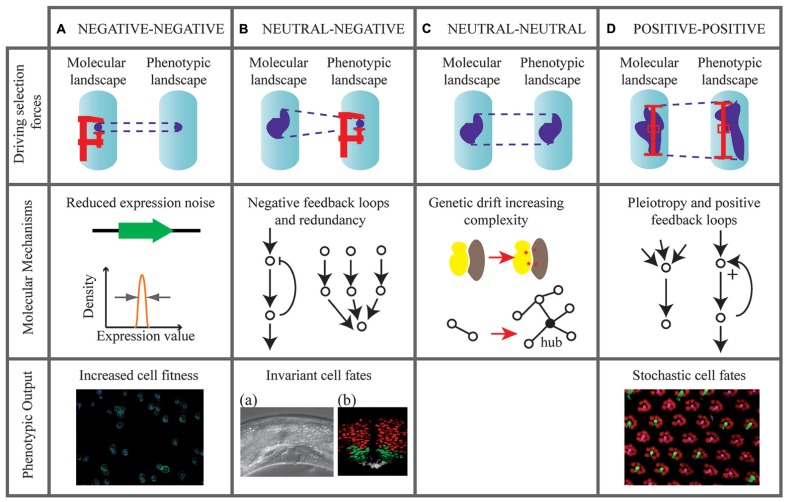
**Evolutionary scenarios that may tune molecular and phenotypic noise.** Four hypothetical scenarios are represented (columns **A–D**).,Evolutionary forces are indicated on diagrams in the upper row. Dark areas in the molecular and phenotypic landscapes indicate the possible states of an individual given its genotype. When the state is variable (high noise) the size of the area is large. Forces that reduce noise (negative selection) are represented by a screw clamp. Forces that maintain noise (positive selection) are represented by a prop. Schemes in the middle row indicate possible molecular architectures involved in these forces. The bottom rows contain relevant examples of phenotypic output (when available). **(A)** Selection forces minimize noise in molecular reactions directly controlling fitness, such as expression of essential genes in yeast. Image: yeast cells dividing. **(B)** Buffering mechanisms allow selection of low phenotypic noise in the presence of molecular fluctuations. Images: (a) Highly invariant vulval development *in C. elegans.* Reproduced from Eisenmann (2005). (b) Invariant localization pattern of Sonic hedgehog targets in the chick embryo (red: Olig2 expression, green: Nkx2.2 expression, white: floor plate cells). Reproduced from Dessaud et al. (2010). **(C)** Noise freely evolves under neutral selection. Genetic drift facilitates the appearance of mutations in protein complexes (red stars), generating network complexity. **(D)** Positive selection for both molecular and phenotypic noise. Image: stochastic distribution of cell fates in the Drosophila eye. Pink: Rh6 photoreceptors. Green: Rh5 photoreceptors. Courtesy of S. Brown and B. Mollereau.

Having said that biological noise is evolvable, can we hypothesize on the evolutionary forces shaping it? As illustrated on **Figure 1**, we describe possible evolutionary scenarios leading to the modulation of molecular and phenotypic noise: (i) how negative selection can minimize molecular noise, (ii) how purifying selection for phenotypic robustness may generate molecular noise, (iii) what neutral forces contribute to noise accumulation, and (iv) how heterogeneity may be positively selected at phenotypic and molecular levels.

## NEGATIVE SELECTION REDUCING MOLECULAR NOISE

Theoretical and experimental work on yeast essential genes strongly support that purifying selection can maintain low biological noise at the molecular level. Intuitively, large or durable fluctuations in the level of an essential protein (i.e., a protein required for yeast cell division) can be deleterious. This was initially suggested by a simple model that predicted low expression noise for essential genes (Fraser et al., 2004). This prediction was demonstrated after Newman et al. (2006) quantified the level of expression noise of 4,000 GFP-tagged yeast proteins and Lehner (2008) correlated noise values with gene essentiality. Another study further showed that correcting for “gene importance” (the loss of fitness caused by deleting the gene) is required before interpreting expression noise determinants at the genomic scale (Zhang et al., 2009). It was even suggested that purifying selection against noise may drive the clustering of essential genes in the genome (Batada and Hurst, 2007). These genomic analyses all showed that negative selection is at play to reduce expression noise of essential yeast genes. They illustrate that the molecular regulations directly involved in fitness (e.g., cell proliferation) are maintained at low noise levels. If these molecules participate to other phenotypic traits, then it is possible that noise of these traits is constrained as well (**Figure 1A**).

## NEGATIVE SELECTION CAN LIMIT PHENOTYPIC NOISE WHILE ALLOWING MOLECULAR NOISE

Phenotypic robustness (defined as the persistence of an organismal trait under perturbation) is a characteristic of many biological systems (Félix and Wagner, 2008). This is particularly illustrated by developmental processes displaying reproducible outcomes and noise minimization (**Figure 1B**). In *C. elegans*, the somatic cell lineage is almost invariant between individuals, due to high reproducibility in cell differentiation (Kipreos, 2005). Similarly, tissue patterning in response to morphogens is highly reproducible between embryos. This was shown in *Drosophila*, where fluctuations in Bicoid concentration were buffered by the slow diffusion of its target Hunchback (Okabe-Oho et al., 2009). It was also shown in vertebrates, where dynamic properties of a regulatory network conferred robustness in the interpretation of the ventral neural tube gradient of Sonic Hedgehog (Dessaud et al., 2010).

More generally, molecular studies showed that many mechanisms can confer robustness of phenotypic outcome in the presence of molecular noise. These include, among others, functional redundancy (Kitano, 2004), negative feedback loops that function as low-pass filters (Becskei and Serrano, 2000), oscillations controlling circadian clocks (Barkai and Leibler, 2000) and frequency-modulation regulations by physical shuttling between cellular compartments (Cai et al., 2008).

Such buffering mechanisms may have apparently paradoxical evolutionary consequences. While maintaining purifying selection for phenotypic robustness, they may relax the selective pressure on molecular noise. If a large genotype space is not expressed as phenotypic variation, genetic mutations have the possibility to evolve neutrally, resulting in the accumulation of cryptic genetic variations. Experimental evidence showed that these buffering mechanisms depend on the environment (Braendle and Félix, 2008) and differ across evolutionary branches (Félix, 2007). Thus, molecular noise may evolve by neutral drift (and possibly accumulate) as a result of purifying selection for robustness in living systems.

## NOISE EVOLUTION UNDER FULLY NEUTRAL SELECTION

Phenotypic traits are not constantly under selective pressures. Situations of small population size or prolonged isolation from environmental constrains let species accumulate mutations that would otherwise be eliminated by purifying selection (Lynch, 2013). When a species experiences such episodes, both molecular and phenotypic noise may freely evolve toward lower or higher levels (**Figure 1C**). Some evidence suggests that this could happen via the evolution of the hubs of protein networks. In populations of reduced size, the accumulation of mildly deleterious mutations was proposed to generate high complexity of protein–protein interactions. Fernández and Lynch (2011) reported evidence from protein structures that such mutations may be compensated by secondary recruitments of interacting partners, which would maintain critical cellular functions. This way, episodes of neutral evolution may increase connectivity in protein networks. Consistently, the constructive neutral evolution theory proposed that, in large populations, increased molecular complexity can be directionally driven without positive selection (Gray et al., 2010; Lukeš et al., 2011). This can generate hubs in protein networks, which can have substantial consequences on noise regulations. If a protein interacts with more partners, the probability to interact actively with any one of them diminishes (competition between partners). This may change the dynamics of molecular regulations by introducing time delays in the formation of complexes, thereby increasing noise. In addition, the structure of proteins is intrinsically plastic (Ward et al., 2004; Uversky and Dunker, 2010), and it was shown that the structure of some hub proteins tend to be more disordered than average (Kim et al., 2008). Thus increasing the number of protein–protein interactions may generate unstable hubs–partner associations, with possible consequences on phenotypic variability. Collecting additional examples of phenotypic noise variation in natural populations is needed to confirm this possibility.

Finally, noise may in return affect evolutionary selection. Using a mathematical model, Wang and Zhang (2011) showed that phenotypic noise can reduce the proportion of the population that is exposed to positive or negative selection. This way, the truly effective population size is reduced, which then favors neutral evolution. Thus, elevated noise may both be a consequence of and a contributor to neutral evolution.

## POSSIBLE POSITIVE SELECTION FOR ELEVATED NOISE

In general, noise is unlikely to be positively selected since it shifts phenotypic traits away from their fitness optimum. However, many examples illustrate how biological systems can exploit noise to their advantage. Anticipative adaptation based on phenotypic heterogeneity has been reported for unicellular organisms. In several cases, observations agreed with an increased geometric mean fitness across generations at the cost of decreasing the arithmetic mean, an investment called “bet-hedging” (Simons, 2011). These include the presence of slow-growing “persister” cells in clonal populations of *E. coli* which survive antibiotic treatment (Balaban et al., 2004). Similarly, clonal yeast populations were shown to contain a minority of slow-dividing cells that could survive extreme heat shock (Levy et al., 2012). Whether such bet-hedging strategies are favored over responsive strategies based on environmental sensing was investigated using simulations. Results suggested that this can be the case if environmental changes are infrequent and unpredictable and if responsive mechanisms have greater cost than benefit (Thattai and van Oudenaarden, 2004; Kussell and Leibler, 2005). However, demonstration of direct selection for heterogeneous phenotype is challenging and has only rarely been achieved. By artificially selecting phenotypic heterogeneity through experimental evolution, Beaumont et al. (2009) showed that positive selection for phenotypic noise is possible in bacteria. Although evidence is even more difficult to collect in the wild, the diversification of timing of *Lobelia inflata* seed germination indicates that such selection may occur in nature (Simons, 2009).

In addition to these phenotypic observations, molecular signatures suggesting positive selection for expression noise were found in the yeast genome. Genes involved in stress response, especially those containing a TATA box, display high noise levels in standard growth conditions (Bar-Even et al., 2006; Lehner, 2010). Genes coding for trans-membrane transporters display both elevated noise in expression and indication of natural selection for it (Zhang et al., 2009), and a wild allele of one of these transporters was associated with increased gene expression noise (Fehrmann et al., 2013).

In multicellular organisms, the contribution of molecular noise to cellular differentiation was proposed long ago by Kupiec (1996) who hypothesized that non-genetic variability could generate a diversity of cellular states on which Darwinian selection could act. This concept is now the focus of active experimental investigations. Clonal populations of hematopoietic stem cells were shown to display heterogeneity associated with variable outcomes in progenitor cell differentiation (Chang et al., 2008). A transient phase of noisy gene expression related to stochastic epigenetic alteration was also associated with cell reprogramming (Buganim et al., 2012). Similarly, the differentiation of T cells into distinct lineages is not strictly deterministic in response to mixed signals (Antebi et al., 2013). A striking example of noise-associated fitness advantage was identified in the *Drosophila* eye, where different photoreceptor types were distributed according to non-repetitive patterns that improved eye perception. This distribution is achieved by the stochastic expression of *spineless* which may be a way to avoid controlling a highly complex deterministic pattern (Wernet et al., 2006). In fact, maintaining fully deterministic processes in all differentiation pathways is probably costly. Driving some cell fate decisions by constrained stochastic processes might be efficient at lower costs, which argues for positive selection for elevated noise.

## DOES H2A.Z CONTRIBUTE TO NOISE EVOLUTION?

The case of H2A.Z is particularly interesting regarding noise evolution. This histone variant has simultaneously received attention from two poorly connected research fields. On one side, biologists working on chromatin regulations have characterized where and how this variant of histone H2A is incorporated in the chromatin. They showed that H2A.Z is highly conserved across evolution, that it is essential to many organisms (Jackson and Gorovsky, 2000; Bönisch and Hake, 2012), and that its presence in the chromatin affects the transcriptional response to environmental cues as well as the efficiency of transcriptional elongation (Hardy et al., 2009; Santisteban et al., 2011; Coleman-Derr and Zilberman, 2012; Weber et al., 2014). In parallel and rather independently to these mechanistic studies, H2A.Z was identified in a screen for genes conferring robustness to phenotypic variations. Using single-cell data from *S. cerevisiae* mutants, Levy and Siegal (2008) found that both H2A.Z and SWR1 (the chaperone that loads H2A.Z on chromatin) have high phenotypic potential, a measure of phenotypic variance when the gene is deleted. The study also showed that H2A.Z interacts physically with many proteins and genetically with many genes via epistasis, characteristics that are predicted to confer buffering capacities (Levy and Siegal, 2008). These authors later reported that deletion of H2A.Z also increased the variance of cell division rates, a typical feature of anticipative adaptation to stress via slow-growing colonies (Levy et al., 2012). The buffering capacity of H2A.Z was also confirmed in mutation accumulation lines, where it was shown to interact epistatically with mutations. This, however, was unrelated to a general increase of mutational robustness (Richardson et al., 2013).

**FIGURE 2:**
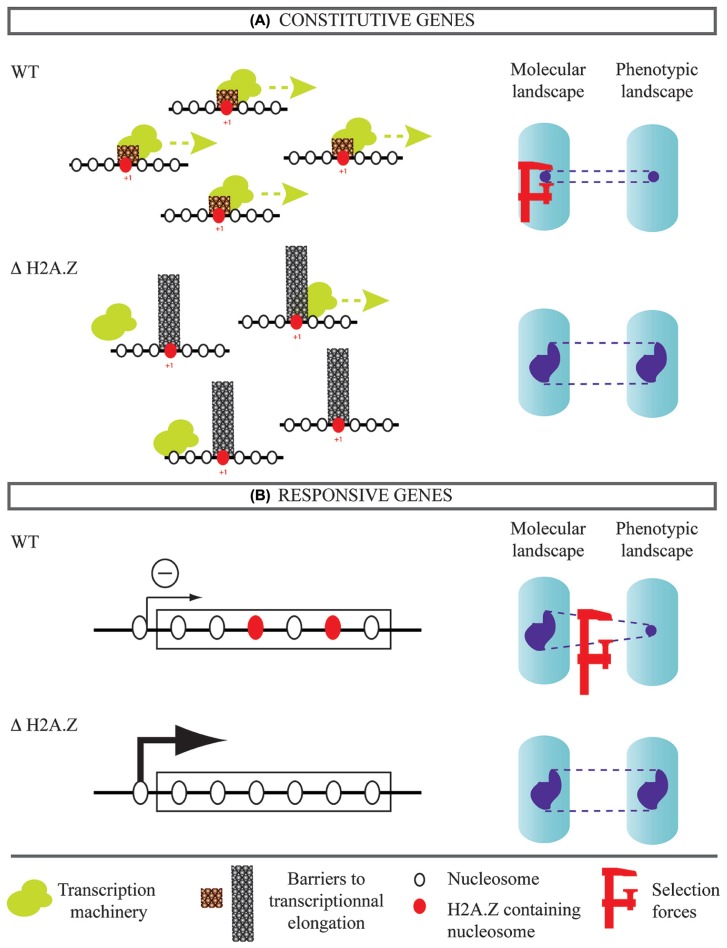
**Possible contribution of H2A.Z histone variant to noise evolvability.** Two classes of genes are considered. **(A)** Genes expressed constitutively. The presence of H2A.Z in the (+1) nucleosome facilitates transcriptional elongation, which may reduce expression noise. In this case, H2A.Z inactivation can increase expression noise of constitutive genes and this would explain the observed phenotypic heterogeneity. **(B)** Genes responding to environmental changes. Presence of H2A.Z in gene bodies correlates with silencing of transcription in the absence of external stimuli. This can enable accumulation of cryptic variations that diversify phenotypes if H2A.Z is inactivated.

How can the known mechanistic roles of H2A.Z explain this phenotypic buffering? We propose two complementary scenarios. The first one concerns genes expressed constitutively (**Figure 2A**). Yeast cells lacking H2A.Z are hypersensitive to drugs or mutations that impair transcriptional elongation (Santisteban et al., 2011). Consistently, H2A.Z was shown to facilitate elongation by decreasing the barrier effect of the nucleosome located immediately downstream the transcription start site (+1 nucleosome; Weber et al., 2014). In parallel, impairment of elongation, such as treatment with 5-azauracil, deletion of yeast TFIIS, PAF1 subunits, or SPT4 were all shown to increase gene expression noise (Ansel et al., 2008). It is therefore possible that the high phenotypic noise among cells lacking H2A.Z results from increased molecular noise, which itself emerges from inefficient transcriptional elongation. The presence of H2A.Z would then contribute to lower both molecular and phenotypic noise. Secondly, H2A.Z may affect noise levels by its repressive action on responsive genes (**Figure 2B**). Incorporation of H2A.Z in the body of genes correlates with reduced expression (Hardy et al., 2009). In *A. thaliana*, H2A.Z across gene bodies is associated with gene functions related to environmental response and could maintain repression in the absence of stimulus (Coleman-Derr and Zilberman, 2012). This may be seen as a buffering mechanism against molecular noise. If a set of genes is maintained silenced in neutral environments because of H2A.Z-dependent repression, the phenotypic expression of their mutations can depend on H2A.Z. This epistasis could generate the observed high phenotypic noise in yeast mutants lacking H2A.Z. Functional studies will be needed to determine if H2A.Z has such a predominant role in the evolution of molecular and phenotypic noise.

## Conflict of Interest Statement

The authors declare that the research was conducted in the absence of any commercial or financial relationships that could be construed as a potential conflict of interest.
